# Elongin A associates with actively transcribed genes and modulates enhancer RNA levels with limited impact on transcription elongation rate *in vivo*

**DOI:** 10.1074/jbc.RA120.015877

**Published:** 2020-12-24

**Authors:** M. Behfar Ardehali, Manashree Damle, Carlos Perea-Resa, Michael D. Blower, Robert E. Kingston

**Affiliations:** 1 Department of Molecular Biology, Massachusetts General Hospital, Boston, Massachusetts, USA; 2 Department of Genetics, Harvard Medical School, Boston, Massachusetts, USA

**Keywords:** Elongin A, RNA polymerase II (Pol II), transcription, transcription elongation, enhancer, RNA polymerase I (Pol I), nucleolus, phase separation, disordered protein, CTD, C-terminal domain, DFC, dense fibrillar component, EloA, Elongin A, eRNA, enhancer RNA, FC, fibrillar center, FDR, false discovery rate, GREAT, Genomic Regions Enrichment of Annotations Tool, IDR, intrinsically disordered regions, LLPS, liquid–liquid phase separation, mEGFP, monomeric enhanced GFP, NDR, nucleosome depleted region, Paf1C, polymerase-associated factor 1 complex, PONDR, predictor of natural disordered regions, TSS, transcription start site

## Abstract

Elongin A (EloA) is an essential transcription factor that stimulates the rate of RNA polymerase II (Pol II) transcription elongation *in vitro*. However, its role as a transcription factor *in vivo* has remained underexplored. Here we show that in mouse embryonic stem cells, EloA localizes to both thousands of Pol II transcribed genes with preference for transcription start site and promoter regions and a large number of active enhancers across the genome. EloA deletion results in accumulation of transcripts from a subset of enhancers and their adjacent genes. Notably, EloA does not substantially enhance the elongation rate of Pol II *in vivo*. We also show that EloA localizes to the nucleoli and associates with RNA polymerase I transcribed ribosomal RNA gene, *Rn45s*. EloA is a highly disordered protein, which we demonstrate forms phase-separated condensates *in vitro*, and truncation mutations in the intrinsically disordered regions (IDR) of EloA interfere with its targeting and localization to the nucleoli. We conclude that EloA broadly associates with transcribed regions, tunes RNA Pol II transcription levels *via* impacts on enhancer RNA synthesis, and interacts with the rRNA producing/processing machinery in the nucleolus. Our work opens new avenues for further investigation of the role of this functionally multifaceted transcription factor in enhancer and ribosomal RNA biology.

Transcription by RNA polymerase II (Pol II) is a multistep process, facilitated by a large number of auxiliary factors that modulate the activity of Pol II (1, 2). In addition to recruitment, pausing of Pol II molecules 20–60 bp downstream of transcription start site (TSS) introduces another regulatory layer, which controls release of Pol II into productive transcription elongation (3, 4, 5), and recent findings point to the generality of this mechanism in regulation of transcription in various metazoan organisms (6). Upon release of Pol II into productive elongation, prebound or recruited transcription elongation factors facilitate passage of Pol II through gene bodies by increasing the elongation rate and/or processivity of Pol II (7, 8, 9). The rate of Pol II elongation has been shown to play a critical role in efficient splicing and development of the organism (10, 11). The elongation rate of Pol II has been shown to vary for different genes with measurements ranging from 1 to 4 kb/min (12).

One of these transcription elongation factors, Elongin A (EloA), was discovered as a biochemical activity that enhances Pol II elongation rate by decreasing the frequency of Pol II arrest and transient pausing in run-off transcription assays (13, 14, 15). EloA forms a heterotrimeric complex with Elongin B and Elongin C, which enhance the complex stability and specific activity of EloA, respectively (15). Internal deletion analysis of EloA identified the C-terminal region of the factor (residues ∼400–773 of rat EloA) as necessary and sufficient for *in vitro* elongation activity (16). *In vivo*, EloA has been shown to play a critical role in optimal gene induction in response to environmental stress and developmental stimuli (17, 18, 19, 20). Moreover, in addition to its role as a transcription elongation factor, EloA is also the substrate-recognition subunit of Cullin-RING E3 ubiquitin ligase complexes, with the largest subunit of Pol II, RPB1 being its main client. Assembly of this complex is triggered upon genotoxic stress and DNA damage, which targets stalled Pol II for degradation (21).

While the role of EloA in transcription elongation by RNA polymerase II has been examined in great detail *in vitro* (14, 15, 16), there are limited reports on the role of Elongin A in transcription *in vivo*. Recently we showed that Elongin A is methylated by PRC2 at K754, a highly conserved residue within a region predicted to form coiled-coil protein interaction domain (16, 22). Here we characterize the *in vivo* function of EloA and show that it localizes to thousands of transcriptionally active protein coding genes in mESC with a strong presence at the TSS/Pol II pause site and downstream of the polyadenylation signal. Surprisingly, evaluation of the Pol II elongation rate upon reactivation of productive elongation revealed that EloA has minimal effects on transcription elongation by Pol II *in vivo*. We also observed that EloA is present at many transcriptionally active enhancers and superenhancers and that its loss results in accumulation of enhancer RNA (eRNA) at a subset of these, as well as elevated levels of nascent RNA at adjacent genes. Furthermore, we show that EloA is associated with RNA polymerase I transcribed ribosomal RNA gene, *Rn45s*, and is present in the nucleoli. EloA is a highly disordered protein, and we demonstrate that purified EloA forms phase-separated condensates *in vitro* and that truncations within the highly disordered region interfere with targeting and localization of EloA to the nucleoli. Our work here uncovers new aspects of EloA function by showing its widespread association with genic and intergenic transcribed regions, its impact on eRNA synthesis, and its presence on rRNA genes and in the nucleolus.

## Results

### Elongin A shows widespread association with actively transcribed genes

**Figure 1:**
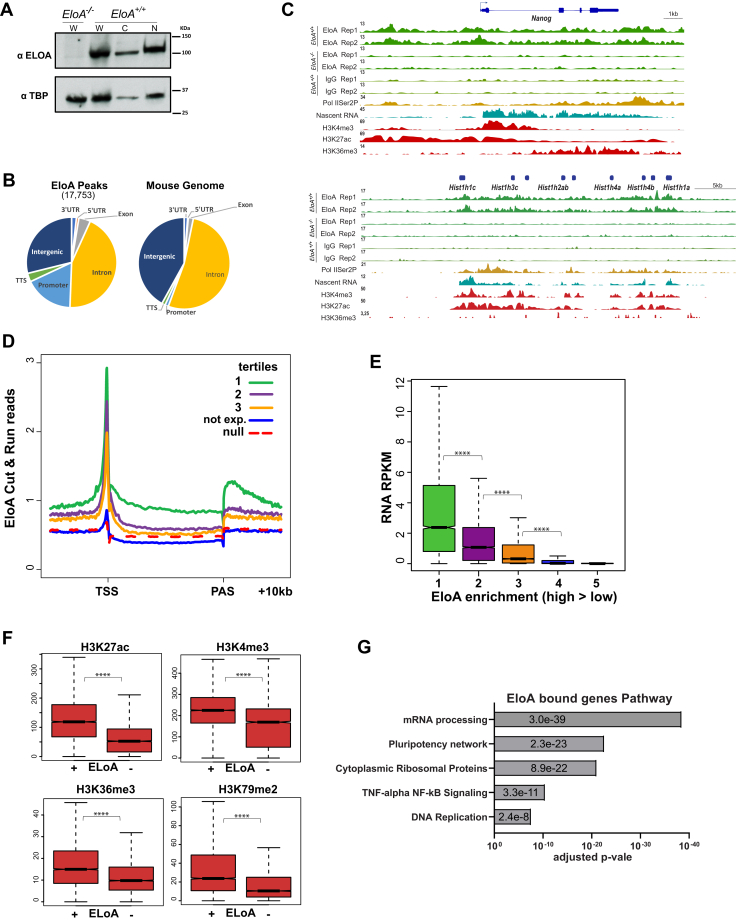
**Elongin A is present at a large number of actively transcribed genes.***A*, immunoblot of whole cell (W), cytoplasmic (C), and nuclear (N) extract of wild-type and *EloA* null mESC, showing nuclear localization of EloA, TBP immunoblot serves as loading and nuclear localization control. *B*, pie chart depicting distribution pattern of 17,753 EloA C&R-seq peaks (FC > 1.5 over null) over known genomic features and its strong association with 5ʹ ends/promoter region of genes. Pie chart on right illustrates distribution of sequences and genomic features across the mouse genome. *C*, EloA Cut&Run-seq genome browser profiles of representative genes, *Nanog* and a histone gene cluster, depicting strong association of EloA with the promoter and post PAS of *Nanog* and widespread association across the transcribed histone gene cluster. *D*, metagene plot of EloA Cut&Run-seq grouped into tertiles based on enrichment level, showing strong TSS and post polyadenylation signal (PAS) enrichment for EloA. *E*, box plot showing positive correlation between EloA enrichment (*x*-axis) and transcription levels (*y*-axis, nascent RNA levels). *F*, box plots showing strong positive correlation between EloA Cut&Run-seq peaks and histone modifications associated with active transcription. *G*, molecular pathways showing strongest enrichment as determined by wikipathways. *p*-values for (*E–F*) derived from Wilcoxon test, ∗∗∗∗*p* < 0.0001.

The association of EloA with Pol II transcribed genes has been tested at a small number of housekeeping and stress response genes in cells expressing tagged EloA (20). To gain a comprehensive understanding of the EloA genome-wide distribution and its role in transcription by Pol II, we carried out ChIP-seq, as well as Cleavage Under Targets and Release Using Nuclease (CUT&RUN) (23, 24) on endogenous EloA in wild-type and *EloA*^*−/−*^ mESCs. We characterized EloA protein levels in wild-type and *EloA* null mES cells by fractionation and by immunoblotting. We found that, as expected, EloA is predominantly a nuclear protein (Fig. 1*A*). While ChIP-seq and Cut&Run-seq peak calling algorithms both identified many of the robustly expressed genes as being bound by EloA, Cut&Run-seq offered a superior signal/noise ratio for the remainder of genes, allowing for a more comprehensive profiling of EloA binding sites (Fig. S1, *A–B*).

CUT&RUN-seq of EloA identified 17,753 peaks (SEACR, FC > 1.5 over null) throughout the genome, more than 70% of which localized to different regions of annotated genes, with a notable bias toward the promoter region (Fig. 1*B*). We established that the EloA CUT&RUN-seq signal is specific by comparing it with that of EloA and IgG CUT&RUN-seq signals in null and wild-type cells, respectively (Fig. 1*C*). EloA was present at the majority of expressed genes (n = 6198, RPKM > 0.5), while 4464 expressed genes were not bound by EloA. Association of EloA at many highly expressed genes exhibited a bimodal distribution pattern, with one strong peak around the TSS/Pol II pausing site and another peak at the 3ʹ-end, downstream of cleavage/polyadenylation signal site (Fig. 1, *C*–*D*). We grouped EloA-bound genes into three classes based on enrichment levels (Fig. 1*D*) and found a strong positive correlation with transcription activity of target genes, as determined by profiling nascent transcription by Bru-seq (Fig. 1*E*). These observations are consistent with an independent report in human DLD1 cells (Wang *et al*., submitted). Expressed genes enriched for EloA also had higher levels of histone modification marks associated with active genes in both the promoter regions (H3K27ac, H3K4me3) and gene bodies (H3K36me3, H3K79me2) (Fig. 1*F*). Molecular pathway analysis of EloA positive genes found that mRNA processing, translation, and pluripotency gene classes were enriched; all categories showing high expression in stem cells (Fig. 1*G*). We also examined nascent transcription by Bru-seq in wild-type and null EloA mESCs and found a small effect on gene expression with 679 upregulated and 198 genes downregulated in null cells (FC > 1.5) (Fig. S1*C*). We conclude that EloA binds to a large number of actively transcribed genes and that loss of EloA has a rather minor effect on gene expression under steady-state conditions, predominantly leading to upregulation of a subset of genes.

In mESCs, roughly half of actively transcribed genes have been shown to be regulated by Pol II pausing early in the transcription process (25). We also noted a positive correlation between EloA enrichment and Pol II pausing, where paused genes displayed elevated levels of EloA near the promoter and around the pause site in comparison with nonpaused genes (Fig. S1, *D–E*). We also noted a moderate decrease in the levels of Pol II-Ser2P in *EloA*^*−/−*^ cells (Fig S1*F*).

### Elongin A is enriched at active enhancers and regulates their transcription

**Figure 2:**
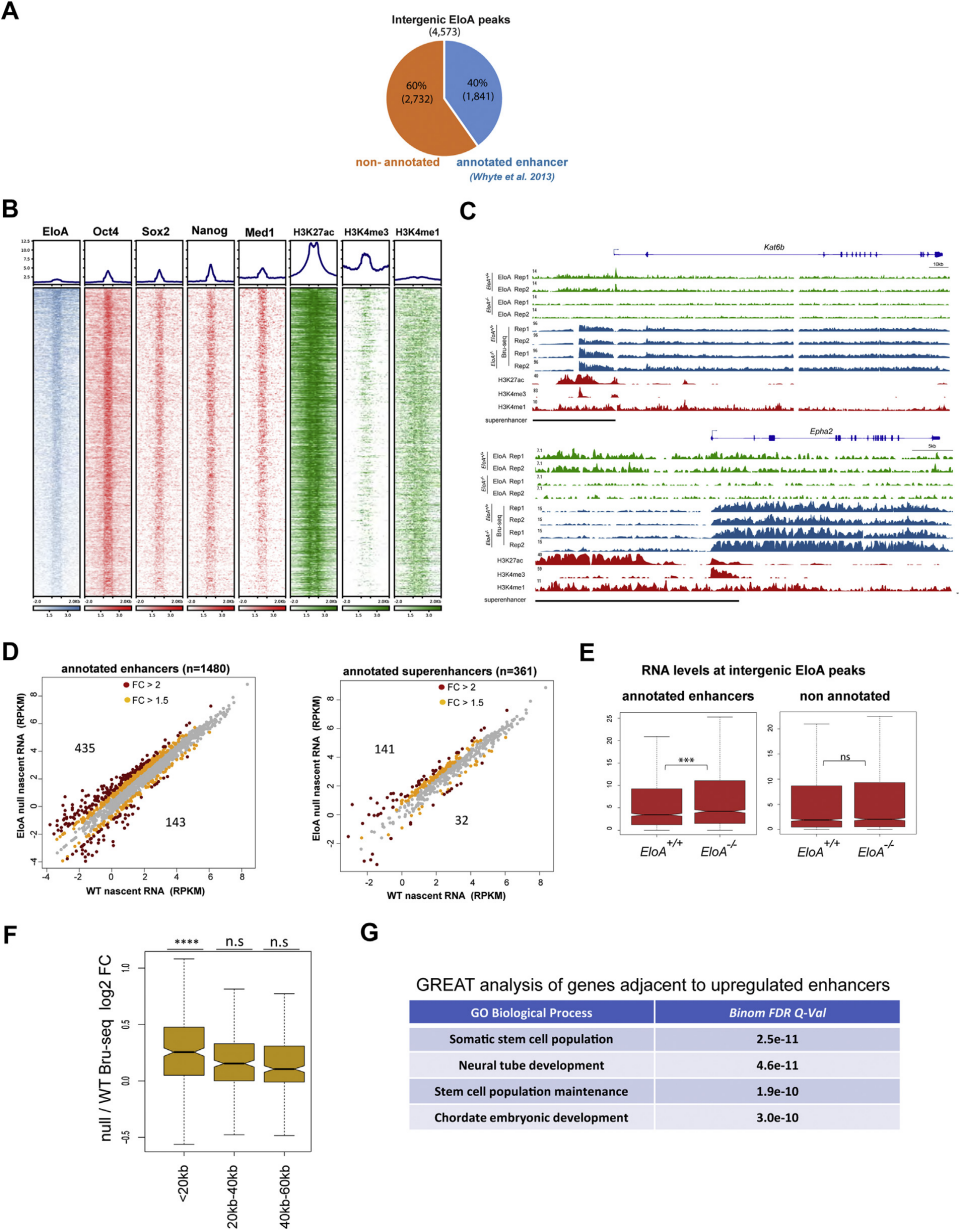
**Elongin A is enriched at transcriptionally active enhancers and superenhancers.***A*, pie chart depicting the degree of overlap between 4573 intergenic EloA peaks and annotated enhancers as described by Whyte *et al*. (28). *B*, heat map of EloA, TFs (Oct4, Sox2, Nanog), mediator subunit, Med1, and histone modifications (H3K27ac, H3K4me3, H3K4me1) occupancy rank-ordered by decreasing EloA enrichment displaying positive correlation between intensity of EloA binding and master TFs, histone H3K27ac and H3K4me3 levels (*n* = 4573). *C*, genome browser screenshots depicting enrichment of EloA and eRNA upregulation at two representative superenhancers upstream of *Kat6b* and *Epha2* genes. *D*, scatter plot showing eRNA nascent RNA levels as detected by Bru-seq in WT and null mESC (left panel: enhancers, right panel: superenhancers). Numbers within the scatterplot denotes number of up or downregulated eRNAs (FC > 1.5). *E*, box plot showing eRNA levels at EloA-bound annotated enhancers and nonannotated sites. *F*, box plot showing the ratio of null over wild-type nascent RNA signal for genes <20 kb, between 20–40 kb and 40–60 kb away from enhancers that are upregulated in *EloA* null cells. *p*-values for (*E–F*) derived from Wilcoxon test, ∗∗∗*p* < 0.001, ∗∗∗∗*p* < 0.0001. *G*, table depicting Genomic Regions Enrichment of Annotations Tool (GREAT) analysis results for identification of gene classes adjacent to enhancers bound by EloA and upregulated in *EloA*^*−/−*^ cells.

Enhancers are mainly distal regulatory elements that harbor binding sites for transcription factors and which, irrespective of orientation, are capable of activating gene expression over large distances (26, 27).We were intrigued by the fact that a quarter of our identified EloA peaks (4573/17,753) were located outside of annotated genes (Fig. 1*B*) and asked whether these intergenic EloA binding sites show any overlap with annotated intergenic enhancers in mESC. Interestingly, out of 4573 intergenic EloA peaks, 40% (1841) overlapped with annotated enhancers, with 361 of these sites localizing to clusters of enhancers, commonly referred to as superenhancers (Fig. 2*A*) (28). We ranked EloA-bound enhancers based on the intensity of EloA enrichment and found a positive correlation between EloA binding and enrichment of Sox2, Nanog, and Oct4, all master transcription factors recruited to enhancers and required for establishment and maintenance of pluripotency in ESCs (Fig. 2*B*). Interrogation of the chromatin landscape at EloA-bound enhancers also revealed positive correlation between EloA levels and intensity of H3K27ac and H3K4me3 at these sites (Fig. 2, *B*–*C*), both modification marks delineating active enhancers (29). Whereas, H3K4me1, an enhancer mark associated with both active and poised enhancers, did not display strong correlation with the intensity of EloA binding (Fig. 2, *B*–*C*).

Active enhancers are transcribed and generate unstable, short-lived RNAs termed enhancer RNAs (eRNAs) (29, 30, 31). Given that EloA is a Pol II transcription factor and that the intensity of EloA binding at enhancers positively correlated with H3K4me3 levels, a mark of transcriptionally active enhancers, we examined the role of EloA in transcription from these distal regulatory elements and measured nascent transcription at enhancers by Bru-seq (±1 kb EloA peak).

Interestingly, our analysis of nascent RNA transcription at EloA-bound annotated enhancers revealed misregulation in eRNA levels at 40.8% (751/1841) of these sites in *EloA* null cells. Strikingly, the majority of these differentially expressed enhancer elements (76.6%) had elevated levels of eRNA transcription in *EloA*^*−/−*^ mES cells (576/751, FC > 1.5) (Fig. 2*D*). In contrast only 3.6% of EloA-bound genic regions show differential expression after loss of EloA (225/6198) (Fig. S1*G*). The observed upregulation of transcription at EloA-bound intergenic loci was statistically significant for only annotated enhancers and not for other intergenic EloA-bound sites (Fig. 2*E*). We also noted that genes in the immediate vicinity (<20 kb) of these upregulated enhancers had higher levels of nascent transcription in *EloA* null cells (Fig. 2*F*). We asked whether these differentially expressed enhancers in *EloA* null cells are adjacent to any specific class of genes and performed Genomic Regions Enrichment of Annotations Tool (GREAT) analysis to address this. Genes adjacent to downregulated enhancers in *EloA*^*−/−*^ did not show a statistically significant correlation with any GO biological process; however, positive correlation was observed between eRNA misaccumulation in *EloA*^*−/−*^ mES cells and genes involved in maintenance of pluripotency as well as neural tube and embryonic development (Fig. 2*G*). In addition to enrichment of EloA at many of these distal intergenic enhancers, the CUT&RUN-seq identified EloA peaks at 469 out of 1912 intragenic enhancers described by Cinghu *et al*. (32) in mESC (Fig. S2, *A–B*). We conclude that EloA is recruited to both intergenic and intragenic enhancers and that its loss results in increased eRNA nascent transcription at several of these sites as determined by Bru-seq. This increased eRNA synthesis correlated with upregulation of adjacent protein coding genes.

### EloA has a negligible effect on transcription elongation by pol II *in vivo*

**Figure 3:**
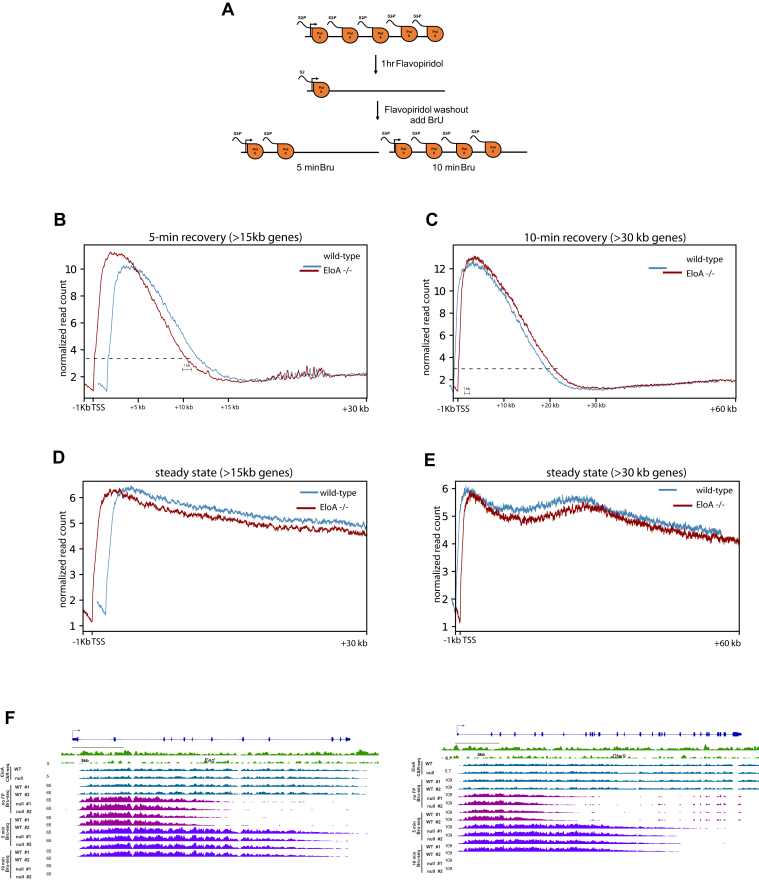
**Examining the role of EloA in transcription elongation by Pol II *in vivo*.***A*, experimental outline for measurement of Pol II elongation rate. *B–C*, line plots showing read density of nascent transcripiton 5-min (*B*) and 10-min (*C*) after release from FP block. Only transcribed EloA-bound genes longer than 15 (5-min) and 30 kb (10-min) were used for measurement of transcription elongation. *Dotted lines* denote the position where nascent RNA signal of Pol II “wave front” approaches background levels for the purpose of estimating the elongation rate (lines are average of two independent experiments). *D–E*, line plots showing nascent transcripiton density (Pol II levels) during steady-state transcription in the absence of FP. Normalized reads: reads per million/bin. *F*, IGV tracks of *Eed* and *Dhx9*, two representative genes showing distribution of nascent transcripts along the gene body 5 and 10-min after release from FP block. The no FP blue tracks depict steady-state levels of nascent RNA.

EloA was initially purified as an activity that stimulates transcription elongation by Pol II *in vitro* (13, 14). However, the extent to which EloA facilitates Pol II elongation *in vivo* has not been explored. To examine the role of EloA in transcription elongation in mESCs, we shut down productive transcription elongation by treating the cells with flavopiridol (FP), a potent P-TEFb inhibitor blocking Pol II C-terminal domain (CTD) Ser2 phosphorylation (33), and measured movement of Pol II within the body of the gene upon removal of the drug. To minimize secondary effects, FP treatment was carried out for only 60 min. After washing out FP, cells were incubated in presence of 5-bromouridine (Bru) for either 5 or 10-min, and nascent RNA was immunopurified and sequenced (Fig. 3*A*). We estimated the position where the read density of nascent transcription approaches background levels on the gene body to be 10–12 and 22–24 kb downstream of the TSS for the 5 and 10-min recovery samples, respectively (intersection of the dotted lines with *x*-axis, Fig 3, *B*–*C*). This corresponds to an elongation rate of 2–2.4 kb/min, which is consistent with previously reported Pol II elongation rates for genes in mESC (12).

The density profile of nascent RNA (transcribing Pol II) immediately downstream of the TSS (+1 kb) at EloA-enriched genes was similar or even slightly higher in *EloA* null cells (Figs. 3, *B*–*C* and S2*C*); however, we noted a discernable, yet modest (∼10%) decrease in the density of reads toward the “wave front” of transcription in the 5-min recovery sample. At 5 min the pioneering wave of Pol II appeared to be lagging by about 1 kb past the 10 kb mark in *EloA*^*−/−*^ cells (Fig. 3*B*). A decrease in nascent RNA signal in null cells was also observed in the 10-min recovery samples (Fig. 3*C*); however, this decrease did not appear more prominent near the leading edge of Pol II transcription (∼+20 kb) but was more notable between the 5 and 10 kb mark. This latter phenotype is not consistent with a substantive role for EloA in modulation of Pol II elongation rate; a more striking difference toward the “wave front” of transcription would be expected. Rather, this suggests that in addition to a potential role in transcription elongation, EloA might also be acting to increase the efficiency of a step upstream of productive elongation such as release form pause, Pol II loading, or initiation. Further, a defect in Pol II processivity would be predicted to cause a drop in the density of transcribing Pol II toward the end of the gene. To examine this issue, we compared the TSS distal to TSS proximal steady-state nascent RNA levels for the same set of genes and did not detect a larger decrease in the density of nascent transcription toward the middle and end of the gene body in null cells (Fig. 3, *D*–*E*). Figure 3*F* depicts movement of Pol II along the body of two representative genes, *Eed* and *Dhx9*, at various time points after transcription reactivation. From these experiments we conclude that the loss of EloA does not cause a significant global change in RNA polymerase processivity. Furthermore, the effect of EloA on Pol II elongation is limited and that the slight decrease in the density of pioneering Pol II wave upon transcripiton reactivation might be mediated at a stage upstream of productive transcription elongation. Our observation regarding the limited effects of EloA on Pol II elongation rate is consistent with an independent study in human DLD1 cells line (Wang *et al*., submitted).

EloA also shows strong enrichment downstream of polyadenylation signal (PAS) (Fig. 1, *C*–*D*). To examine whether EloA plays role in Pol II termination, we also looked at the levels of nascent RNA up to 10 kb downstream of transcripiton end site and noted no difference in the density of Bru-seq signal (Pol II levels) between WT and *EloA* null cells (Fig. S2*F*).

### Characterization of EloA binding partners

**Figure 4:**
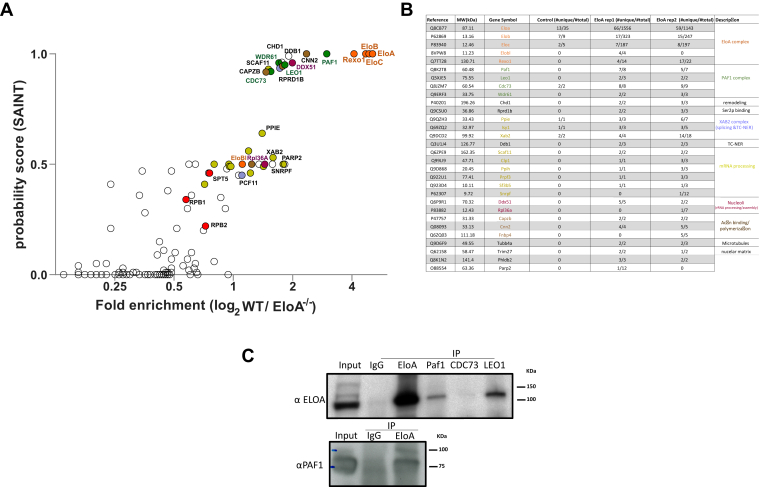
**Characterization of Elongin A proteome interaction network.***A*, affinity purification mass spectrometry (Co-IP/MS) analysis of native Elongin A binding partners from mESC nuclear extract showing major interaction between EloA complex (*orange*) and Paf1complex subunits (*green*) as well as pre-mRNA processing and splicing factors (*olive green*), and cytoskeletal filament interacting proteins (*brown*). *x*-axis denotes log_2_ WT/null EloA enrichment and *y*-axis displays SAINT (significance Analysis of INTeractome) score. *B*, total peptide count from each pull-down for a selected group of factors. Control IP was performed using EloA antibody on MEF depleted EloA null mESC. Leftover MEF cells have likely contributed to EloA peptide count in control sample. *C*, IP immunoblot experiment for validation of interaction between EloA and PAF1 and other Paf1 complex components. List of all identified proteins and their corresponding peptide count, and the raw data are accessible under ProteomeXchange ID: PXD022138.

To better understand the biological processes that EloA may be participating in, we sought to identify the protein interaction network of EloA. We carried out coimmunoprecipitation mass spectrometry (Co-IP/MS) analysis on endogenous EloA from MEF-depleted mESC nuclear extract (Fig. 1*A*). As expected, EloA showed the strongest interaction with EloC and EloB, the other subunits of the heterotrimeric Elongin complex (Fig. 4*A*), as well as the putative RNA exonuclease Rexo1 (EloA-BP1), a previously reported major binding partner of EloA (34). Our results also revealed strong binding between Elongin A and PAF1, LEO1, CDC73, and WDR61(SKI8) (Fig. 4, *A*–*B*), all components of the multifunctional polymerase-associated factor 1 complex (Paf1C), which is involved in regulation of various stages of transcription by Pol II (35). We validated these interactions by IP-Immunoblot experiments (Fig. 4*C*). This observation is concordant with recent reports where pull-down of Paf1C identified EloA as a major interacting partner of the complex in murine myoblast and ESC (7, 36). The protein interactome analysis uncovered evidence for physical interaction between EloA and chromatin remodeling factor CHD1, as well as pre-mRNA processing factors, in particular splicing factors (Fig. 4, *A*–*B*). There were also interactions between EloA and nucleolar proteins (Ddx51, Rpl36a), a finding relevant to the studies of nucleoli we describe below. We found multiple components of the Xeroderma pigmentosum group A (XPA)-binding protein 2 (XAB2) complex (XAB2, ISY1, and PPIE), a multifunctional complex involved in splicing and transcription-coupled DNA repair among EloA interacting proteins (37). The IP was performed on nuclear extracts without including the solubilized chromatin fraction, which in part explains lower levels of interaction with Pol II and other transcription factors (Fig. 4*A*, *red dots*).

### EloA is present at sites of RNA polymerase I transcription

**Figure 5:**
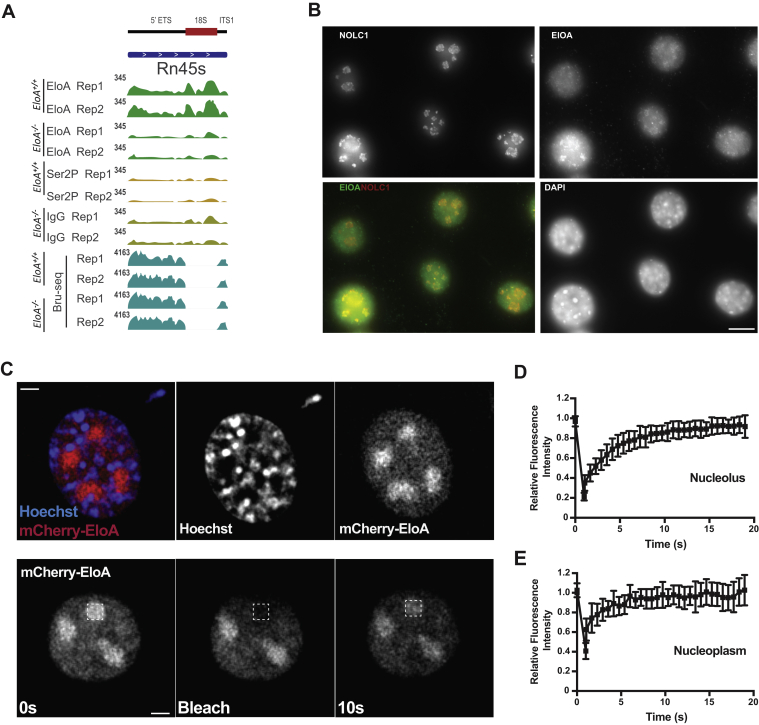
**Elongin A associates with Pol I transcribed ribosomal genes.***A*, IGV genome browser screen shot of Pol I transcribed 45S preribosomal RNA, *Rn45s* located on chromosome 17 showing enrichment of EloA as determined by Cut&Run-seq. Background level of EloA in null mESC serves as a control for specificity of EloA enrichment. *B*, coimmunofluorescence micrograph (*z*-stacks) of formaldehyde fixed 3T3 cells showing colocalization of EloA and nucleolar/Cajal bodies marker protein NOLC1. Scale bar, 11 μm. *C*, FRAP analysis of nucleolar mCherry-EloA demonstrating the dynamic nature of EloA within this organelle. Top panel: Live cell image of mCherry-EloA (*red*) in 3T3 cells and DNA (Hoechst, blue). Bottom panel: Bleached cells imaged for fluorescence recovery. Images of prebleach, bleached, and 10 s post recovery are shown from representative cell. Scale bar: 3 μm. *D* and *E*, relative fluorescence intensity between bleached (*squares*) and nonbleached reference areas plotted over time. Recovery analysis of nucleolar (*D*) and nucleoplasmic (*E*) signal showing distinct recovery profiles. n ≥ 20 from two independent experiments. error bars denote SD.

The 45S pre-rRNA is transcribed by RNA polymerase I (Pol I) and processed into 18S, 5.8S, and 28S rRNAs. Interestingly, we observed association of EloA with segments that map to *Rn45s*, a 45S coding gene on chromosome 17. The EloA CUT&RUN-seq signal was specific for wild-type mES cells and close to IgG background levels in *EloA* null cells, and we did not detect enrichment of Ser2P Pol II at this rRNA gene site (Fig. 5*A*). We carried out coimmunofluorescence microscopy on 3T3 fibroblast cells and observed colocalization between EloA and NOLC1 (Nopp140) (Fig. 5*B*), a nucleolar factor, present at the fibrillar center (FC) and dense fibrillar component (DFC), which are sites for rRNA transcription and processing, respectively (38, 39). This finding is in agreement with our observation that nucleolar factors Rpl36a and DDX51 were identified as interacting partners of EloA (Fig. 4, *A*–*B*).

Nucleolar proteins have been shown to display rapid exchange dynamics between the nucleolus and the nucleoplasm (40), and a subset of tested nucleolar proteins possess liquid-like *in vivo* dynamics related to the known liquid–liquid phase separating properties of nucleoli (41). We characterized the nucleolar dynamics of EloA using a system in which monomeric red fluorescent protein tagged EloA (mCherry-EloA) was expressed from a doxycycline (dox)-inducible construct (Fig. S3*B*). Consistent with the immunofluorescence results on endogenous EloA, mCherry-EloA was present in the nucleolus in addition to the nucleoplasm (Fig. 5*C*, upper panel). We examined the exchange kinetics of mCherry-EloA in 3T3 cells to determine whether it showed exchange characteristics similar to that of other nucleolar proteins. Fluorescence recovery after photobleaching (FRAP) analysis of EloA molecules in the nucleoli showed rapid recovery of photobleached EloA (10–15 s) post photobleaching (Fig. 5, *D*–*E*), demonstrating the dynamic nature of EloA in the nucleolus. Nucleolar EloA recovery was slower compared with the nucleoplasmic mCherry-EloA recovery profile (5 s), which might represent the diffusion dynamics of nucleoplasmic EloA. The slower recovery rate of nucleolar mCherry-EloA might reflect interaction of EloA with binding partners within the nucleolus. We conclude that the nucleolar fraction of EloA possesses dynamics in cell culture that are consistent with those of previously described nucleolar proteins (40, 42).

### Elongin A phase separates and forms condensates *in vitro*

**Figure 6:**
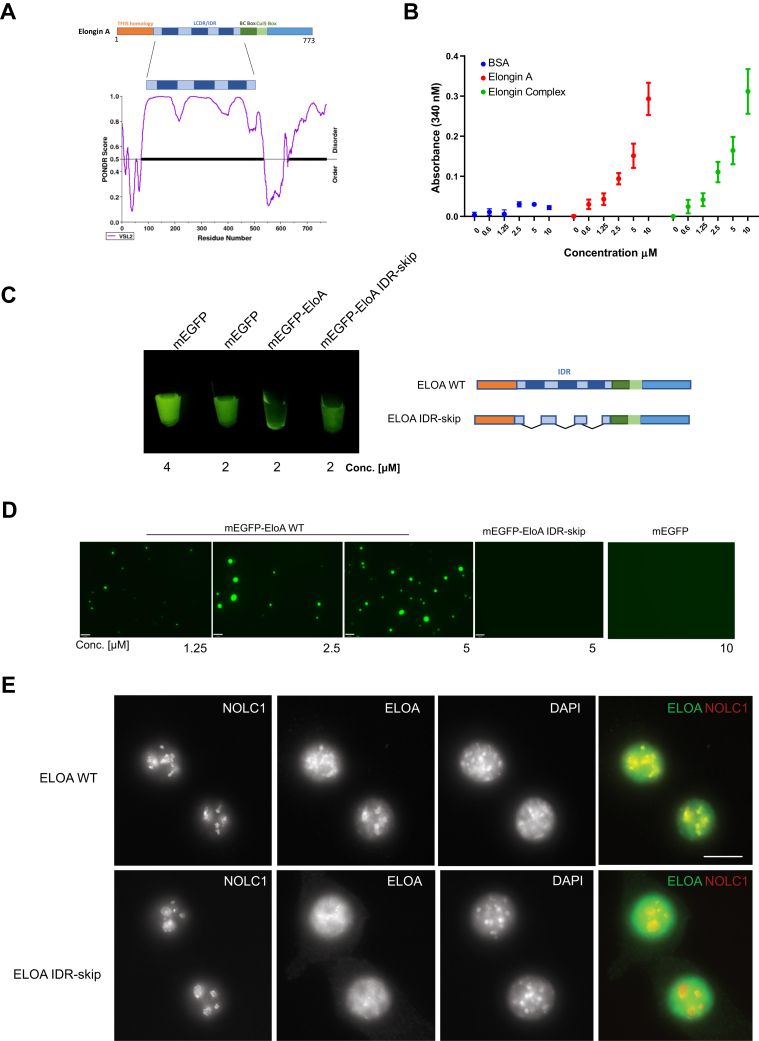
**Elongin A forms phase-separated condensates *in vitro*.***A*, *Top*, schematic representation of Elongin A domains with the highly disordered stretches of amino acids in the low complexity disordered region LCDR (IDR) depicted in *dark blue*. *Bottom*, predictor of natural disordered regions (PONDR) graph showing intrinsic disorder regions of EloA using the VSL2 algorithm. *B*, turbidity assay illustrating formation of turbid solution by purified EloA and EloA complex in a concentration dependent manner at 100 mM KCl buffer concentration, BSA used as negative control. *n* = 3, error: SD. *C*, spin-down assay demonstrating formation/separation of protein condensates by mEGFP-EloA, mEGFP-EloA IDR-skip truncation construct remains predominantly in solution. Right: illustration showing truncation within the IDR for the EloA IDR-skip construct. *D*, representative micrographs showing formation of phase-separated condensates by mEGFP-EloA WT at increasing concentrations in the presence of PEG, while mEGFP-EloA IDR-skip fails to form these droplets under the same conditions. Scale bar: 6 μm. *E*, truncation of EloA IDR interferes with formation of EloA nucleolar condensates *in vivo*. Micrograph of 3T3 fibroblast cells upon 50–100 ng/ml Doxycycline induction of EloAWT (top) and EloA IDR-skip (bottom) constructs. Images represent *z*-stacks from formaldehyde fixed cells immunostained using EloA antibody (*green*), nucleolar marker, NOLC1 (*red*) and DNA (DAPI, *blue*). Scale bar: 11 μm.

The liquid–liquid phase separation properties (LLPS) of the nucleolus have been proposed to be driven in part by proteins with large intrinsically disordered regions (IDR) that facilitate multivalent interactions and formation of phase separated condensates (41). Many of these proteins also interact with RNA, and this interaction is thought to play role in compartmentalization of this highly dynamic nuclear body (41, 43, 44). Analysis of Elongin A protein sequence using predictor of natural disordered regions (PONDR) revealed that 82% of EloA is predicted to be disordered (Fig. 6*A*). Given the localization of EloA in the nucleolus, we examined the ability of EloA to exhibit properties of phase separation *in vitro* and whether this might be related to nucleolar localization.

To determine whether EloA has any of the properties of phase separating proteins, we purified full-length EloA and demonstrated that EloA forms a turbid solution in a concentration-dependent manner (Fig. 6*B*). We also expressed and purified monomeric enhanced GFP (mEGFP) tagged EloA (mEGFP-EloA WT) (Fig. S3*A*) and found that upon centrifugation, mEGFP-EloA separates from solution and forms condensate pellets (Fig. 6*C*). To examine whether the IDR region of EloA plays a role in phase separation, we purified a truncated EloA construct where three highly disordered stretches of amino acids within the IDR were deleted (mEGFP-EloA IDR-skip). This truncation interfered with pellet formation and the protein predominantly remained in solution (Fig. 6*C*). Furthermore, using fluorescence microscopy, we demonstrated that, in the presence of a volume excluder, mEGFP-EloA forms protein-rich spherical droplets in a concentration-dependent manner (Fig. 6*D*), whereas the IDR truncation mutant failed to form these protein-rich foci and had diffuse distribution even at the higher tested concentration (Fig. 6*D*). We conclude that EloA is able to form phase-separated condensates *in vitro* and that truncations within the highly disordered stretches of EloA IDR appear to interfere with phase separation properties of the protein. We noted the presence of a smaller protein band in our mEGFP-EloA IDR-skip protein preparations that might reflect stability issues of the truncated protein and may have impacted our *in vitro* results (Fig. S3*A*, right panel).

Next, we introduced doxycycline (Dox)-inducible wild-type and IDR-skip mutant constructs in *EloA* null 3T3 fibroblast cells using lentiviral transduction and determined the doxycycline concentration required for expression of the constructs at the endogenous levels (Fig. S3, *C–D*). Similar to endogenous EloA (Fig. 5*B*), Dox-induced wild-type EloA localized to the nucleoli (Fig. 6*E*, upper panel). Interestingly, EloA IDR-skip appeared to be partly excluded from the nucleoli and displayed a more diffuse nuclear distribution pattern (Fig. 6*E*, lower panel). We conclude that the highly disordered stretches of amino acid within EloA IDR play a role in localization of EloA to the nucleolus. This might reflect direct effects of these disordered domains on phase separation, as noted above, and also could be influenced by possible impact of this mutation on interactions with other proteins such as EloB and EloC, although the interacting BC box remains intact in the truncated EloA construct.

## Discussion

The role of EloA in transcription elongation by Pol II on DNA templates *in vitro* has been examined in great detail (14, 15, 16); however, its role in Pol II transcription *in vivo* has remained underexplored. Here we show that EloA is enriched at thousands of transcriptionally active genes in mESC and that it shows a strong association with TSS and sites of Pol II pausing (Fig. 1). This is consistent with recent findings from an accompanying report (Wang *et al*., submitted), as well as previous immunofluorescence (IF) analysis showing greater overlap between EloA and Ser5P form of Pol II, as opposed to Ser2P (20). We also observed that loss of EloA resulted in disruption, mostly upregulation, of a few hundred genes in mESC, which is in part in agreement with previous reports (45) and recent findings in DLD1 cells (Wang *et al*., submitted). While loss of EloA has little effect on steady-state gene expression, it has been shown to play a critical role in transcription induction in response to thermal and genotoxic stress (17, 19, 20), as well as activation of developmental genes in response to developmental cues (18, 46). Beyond its role as a transcription factor that is primarily associated with TSS and sites of Pol II pausing, EloA might also be involved in certain steps of signal transduction upon encountering these external stimuli. Future genome-wide experiments studying the role of EloA in response to external stimuli should shed more light on the function and importance of this factor in these events.

Notably, we observed that EloA is enriched at intragenic and intergenic enhancers, which are regulated by Pol II pausing (29, 32) and that its loss in ES cells results in accumulation of transcripts from many of these annotated enhancers and superenhancers, as well as elevated pol II transcription of adjacent genes (Fig. 2). Recently it was shown that PAF1 negatively regulates enhancer transcription *via* controlling the release of paused Pol II and that its loss leads to upregulation of eRNA transcription (47). Moreover, other studies indicated that the integrator complex plays a role in processing and regulation of eRNA transcription and that its depletion results in accumulation of unprocessed eRNA and increased productive elongation (48, 49). EloA shows strong physical interacts with both of these aforementioned complexes (this work and Wang *et al*., submitted), and we observed that its loss also leads to accumulation of nascent eRNA. Future experiments that examine the extent of interplay between EloA, PAF1, and the integrator complex would further elucidate the mechanism of enhancer RNA transcription and regulation.

We observed an increase in the level of nascent transcription immediately downstream of genic TSS (+0.5 kb), as well as upstream of divergent antisense transcription units in null cells (Fig. S2, *C–D*), but not toward the TES in null cells (Fig. 3, *D*–*E*). One explanation for this could be that EloA negatively regulates Pol II pausing and residence time of paused Pol II and that its loss may lead to rapid turnover of paused Pol II and an increase in nascent transcription immediately downstream of the TSS. We also noted strong enrichment of EloA over the promoter and regulatory regions of actively transcribed genes (Figs. 1*D* and S2*E*), suggesting that EloA might have preference for the upstream nucleosome depleted region (NDR), which harbors regulatory elements. The presence of EloA at these sites might also reflect its role in transcription from upstream divergent (antisense) sites, as loss of EloA also leads to upregulation of transcription from the negative strand at upstream divergent transcripiton sites (Fig. S2*D*). This observation indicates a possible role for EloA in regulation of antisense transcription.

Under our experimental setting, we estimated the global elongation rate of Pol II to be in the range of 2–2.4 kb/min. This is concordant with previously reported Pol II elongation rates in mESC by studying the “receding wave” of Pol II upon transcription shutdown (12). Elongin A was identified as an activity that significantly stimulates transcription elongation by Pol II *in vitro*. However, we observed a rather modest (∼10%) drop in the elongation rate of Pol II in *EloA* null cells upon reactivation of transcription (Fig. 3). Moreover, the decrease in nascent RNA levels in *EloA* null cells in the 10-min sample did not appear accentuated around the “pioneering wave” of transcription and was more prominent upstream. This is not consistent with the phenotype expected for a factor that modulates elongation rate of Pol II (Fig. 3*C*). Therefore, it is possible that EloA also plays role in steps upstream of productive elongation. There could be several explanations for this discrepancy between *in vitro* and *in vivo* effects of EloA on transcription elongation rate. First, *in vitro* transcription assays are performed at limiting NTP concentrations and monovalent salt concentrations that are below physiological levels (50). The latter could drive condensate formation by highly disordered proteins such as EloA and induce macromolecular crowding, which on top of the elongation activity of EloA might further increase rates of transcription by Pol II *in vitro*. Second, our experiments were carried out in *EloA* null cells, which might over time adjust and adapt to loss of EloA. Moreover, redundancy and compensation by other transcription factors in the highly complex chromatin environment within living cells could also account for this modest effect on transcription elongation. Third, it is plausible that the role of EloA in stimulating transcription elongation is not global and only limited to a subset of genes with unique regulatory features (*e.g.*, stress response genes).

Interestingly, we also provide evidence for the potential of EloA to be involved in the production of rRNA. We show that EloA localizes to nucleoli and that it is present at the ribosomal RNA gene, *Rn45s* (Fig. 5). Physical interaction with rRNA processing factors provided additional support for involvement of EloA in rRNA transcription/processing (Fig. 4*B*). Our results also show that EloA is capable of forming phase-separated condensates *in vitro*, a feature shared among tested nucleolar proteins (41, 43). Lastly, we show that deletion of three highly disordered stretches within the IDR of EloA interferes with phase separation of EloA *in vitro* and its localization to the nucleolus (Fig. 6*E*). This is consistent with a study showing truncations within the IDR of EloA interfere with localization of EloA to sites of DNA damage (51). These data are consistent with an expanded range of activities for EloA in the nucleus that go beyond RNA Pol II transcription. In summary, we observe broad association of EloA with genes, and a more limited impact on steady-state transcription, especially transcription elongation, than expected based upon *in vitro* activity of this protein. The behavior we characterize is consistent with a broad-based function that tunes nuclear processes at a large set of genes

## Experimental procedures

### Cell culture

Elongin A null (*EloA*^*−/−*^) and wild-type mES cells were kind gift of Dr Teijiro Aso (Kochi Medical School) (46). Cells were grown as described elsewhere (22). In brief, gelatinized plates (0.2%) were seeded with mouse embryonic fibroblast (MEF) as feeder layer and cells were grown in ES cell media containing DMEM knockOut medium, 15% hyclone FBS, 1× GlutaMAX, 1× NEAA, 1× pen/strep, 2 × 10^3^ Units/ml of mLIF (Millipore), 55 μM 2BME, 1 μM and 3 μM of PD0325901 and CHIR99021, respectively. NIH/3T3 (ATCC CRL-1658) mouse embryonic fibroblast cells were grown in DMEM supplemented with 10% FCS.

### Cut&Run-seq

Cut&Run-seq was carried out as described by the Henikoff lab with the following modifications: Mouse ES cells were cross-linked with 1% formaldehyde in PBS for 10 min and quenched with 0.125 M glycine for 5 min, cells were pelleted and washed once with PBS, spun down, resuspended in freezing media (DMEM, 10% DMSO), placed in a styrofoam container, and frozen O/N (this step was included to minimize cell membrane rupture and DNA breakage). On the day of the experiment, cross-linked cells were thawed and washed once with PBS and twice in cut&run wash buffer (20 mM HEPES pH7.5, 150 mM NaCl, 0.1% Tween20, 0.1% BSA, 1 Roche complete EDTA-free protease inhibitor tablet/50 ml), Tween20 was included in the wash buffer to minimize concanavalin A magnetic bead aggregation in downstream steps. Next, 5 × 10^6^ cells were resuspended in 950 μl of wash buffer. Fifty microliters of BioMag-Plus Concanavalin A beads (Polysciences Inc) was also washed gently twice with binding buffer (20 mM HEPES pH 7.9, 1 mM CaCl_2_, 1 mM MnCl_2_) and resuspended in binding buffer at the initial volume (50 μl). Concanavalin A beads (50 μl) were added to 5 × 10^6^ cells in 950 μl of wash buffer and incubated rotating for 15 min at RT. The cells were divided into five 200 μl aliquots for each antibody used (1 × 10^6^ cells/antibody) and after removal of wash buffer, 50 μl of ice cold antibody buffer (wash buffer containing 0.05% Digitonin and 2 mM EDTA) containing 0.5 μg of antibody was added to 1 × 10^6^ bead-bound cell pellet, and the mixture was gently resuspended by pipetting up and down 25 times. Resuspended beads were placed on a vortex at the lowest setting and incubated at room temperature for 15 min. For the EloA cut&run experiment using goat anti EloA (SCBT, R-19), a bridging rabbit anti-goat antibody was used for efficient binding to protein A. Cells were washed once after primary antibody incubation with 1 ml of Digitonin buffer (Wash buffer containing 0.05% Digitonin) resuspended in 50 μl of digitonin buffer with 0.5 μg of rabbit anti-goat IgG (rabbit anti-goat IgG [ab6697]). Left on the lowest setting of the vortex and incubated at RT for 15 min. Next, permeabilized, antibody-bound cells were washed once in digitonin buffer containing 1 mM EDTA, and 50 μl of digitonin buffer containing Protein A-MNase (pA-MN) at a final concentration of 700 ng/ml was added to the pellet, resuspended, and incubated at room temperature for 15 min. Cells were washed twice with digitonin buffer containing 1 mM EDTA. Next, 150 μl of ice cold digitonin buffer was added to the pA-MN bound cells, resuspended, and digested on ice (0 °C) for 30 min after addition of CaCl_2_ to a final concentration of 4 mM. Hundred microliter of 2× stop buffer was added as described in (24) and incubated on thermomixer for 15 min at 37 °C to release the cleaved DNA fragments into solution. The mixture was spun down in a cold tabletop centrifuge at max speed for 5 min, and 2 μl of proteinase K was added to the supernatant eluate. The mixture was incubated at 65 °C for 4 h, and DNA was extracted by using ChIP DNA Clean & Concentrator (Zymo research). Purified DNA was used for library preparation as described elsewhere (52).

### Data processing and bioinformatic analysis of cut-and-run-seq

All experiments were performed in duplicate. Sequencing reads were aligned to the mm10 genome using bowtie2 (53) and filtered using samtools (54) to keep uniquely aligned reads. Genome browser tracks were generated using Homer v4.10.3 (55) and visualized in IGV (56). Peaks were called in each replicate using SEACR (57) using IgG and “relaxed” parameters. Peaks in wild-type cells that were atleast 1.5-fold over null cells were used further for analysis. Homer's annotatePeaks function was used to find the distribution of peaks in the genome (Fig. 1*B*). The same annotations were used to calculate the proportions of features in the mouse genome for comparison. Deeptools v3.3.0 (58) was used to make average profile line plots. HOMER's annotatePeaks function was used to calculate signals for boxplot and R was used to make boxplots. EloA-bound genes were defined as having at least one EloA peak from TSS-0.5 kb to TES. Unique transcripts for each gene were chosen based on the start of the Bru-seq signal. Intergenic peaks were defined as peaks not overlapping genes (TSS-0.5 kb-TES).

### Nascent RNA sequencing (Bru-seq)

Bru-seq was used as a readout for the position and levels of Pol II on gene bodies to determine the elongation rate of Pol II. Moreover, eRNAs are highly unstable and are rapidly degraded upon transcription, which makes them hard to detect using conventional RNA sequencing methodologies. Bru-seq also allowed for better enrichment and detection of eRNA species that would have been hard to detect using conventional RNA sequencing. For nascent RNA analysis experiment, ES cells were depleted of MEF and plated on gelatin-coated 100 mm plates for 3–4 h. For the steady-state nascent RNA level study, 5-Bromouridine (BrU) (2 mM final conc) was added to cells for 10 min. For the elongation rate analysis using flavopiridol (FP), FP (Cayman Chemical, Inc) was added at a final concentration of 1 μM for 60 min. Plates were washed with PBS and incubated in media containing 2 mM BrU for 5 or 10 min. Plates were washed once with PBS and total RNA was isolated by addition of 4 ml of Trizol. Bru-seq was performed as described previously (22, 59) on 100 μg of total RNA from specified mESC cells. Five-hundred microliters of mouse anti-BrdU antibody (200 μg/ml, SCBT, sc-32323) was coupled to 1 ml of PBS-DEPC washed Dynabeads M-280 sheep anti-mouse IgG (11201D). Coupling was done in Bru-seq buffer (PBS-DEPC, 0.1% BSA, 0.05% Tween20), and yeast tRNA was added to a final concentration of 500 ng/μl. Coupling was carried out for 3 h at 4 °C. Coupled antibody was washed three times and stored in 1 ml of Bru-seq buffer. Prior to enrichment of nascent RNA, two consecutive rounds of mature mRNA depletion were carried out on 100 μg of total RNA using Oligo d(T)_25_ Magnetic beads (NEB Inc) according to manufacturer's protocols. We also generated a pool of BrU-incorporated *in vitro* transcribed *Drosophila* spike in RNAs using 5-Bromouridine 5′-triphosphate (B7166-5MG) and MEGAscript T7 Kit (AM1333) according to manufacturer's protocol. Names of *Drosophila* genes and the primer sequences used for synthesis of the templates are provided in Table S1. The *in vitro* transcribed spike in RNAs were pooled at varying concentrations and added to the total RNA from each sample. RNA immunoprecipitation and washing steps were carried out in the Bru-seq buffer as described above and previously (22). In brief, 75 μl of antibody coupled beads was incubated with preheated (95 °C for 1 min followed by rapid cooling on ice) mature mRNA depleted total RNA for 90 min at RT in the dark. After the final washing step (four times total, 5–10 min each), the enriched RNA bound to beads was isolated using Trizol extraction. Ribosomal RNA was depleted using NEBNext rRNA Depletion Kit (NEB), and first strand and directional second strand DNA synthesis was carried out using NEBNext Ultra II first, and directional second strand synthesis modules (NEB Inc), respectively. Library preparation was carried out as described previously (52).

### Bru-seq data processing

All experiments were performed in duplicate. Paired end sequencing reads were aligned to the mm10 and dm3 genomes using bowtie2 and filtered using samtools to keep uniquely aligned reads. Reads from the positive and negative strand were separated using samtools. Browser tracks were generated using Homer v4.10.3 and visualized in IGV. Reads in whole gene of Refseq annotated genes were counted using featureCounts v1.6.1 (60). edgeR (61) and R v3.3.2 were used to normalize reads and calculate RPKMS and fold changes. All further calculations and figures were made using R and Deeptools.

### ChIP-seq

ChIP was carried out on 2.5-5 × 10^6^ cross-linked mESC cells as described previously (22).

### ChIP-seq data processing

All experiments were performed in duplicate. Sequencing reads were aligned to the mm10 genome using bowtie2 and filtered using samtools to keep uniquely aligned reads. Peaks were called in each replicate separately using HOMER's findPeaks function for broad peaks. Peaks that were 1.5FC over null in both replicates were used for the overlap analysis with cut-and-run peaks.

### Turbidity assay

EloA and EloA complex that were purified and described previously (22) were used for the turbidity assay. Proteins were diluted to the described concentrations in the following buffer: 20 mM HEPES pH 7.9, 10% glycerol, 1.5 mM MgCl_2_, 100 mM KCl and placed in clear-bottomed 384-well plates. Absorbance was measured at 405 nm using a Spectramax M3 plate reader. Values are average of at least three samples and errors denote standard deviation.

### Centrifugation assay of mEGFP tagged proteins

mEGFP or mEGFP-ELOA proteins were purified as described above and brought to the specified concentration in 0.2 ml microcentrifuge tubes with the buffer used for turbidity assay. Samples were spun down at 2000*g* for 5 min and visualized under Safe Imager 2.0 Blue Light Transilluminator.

### Fluorescence microscopy of protein condensates

Recombinant mEGFP and mEGFP-EloA were diluted to the specified concentration in 20 mM HEPES pH 7.9, 10% glycerol, 1.5 mM MgCl_2_, 100 mM KCl, and 10% PEG-6000 (crowding agent) and placed on a glass slide and covered with a coverslip. Images were acquired using the 100× oil objective on Nikon 90i Eclipse epifluorescence microscope equipped with an Orca ER camera and velocity imaging software (Perkin Elmer).

### Immunofluorescence

Cells were grown on coverslips and fixed with 1% formaldehyde for 10 min, washed with PBS, and permeabilized with 0.2% Triton X-100 in PBS for 15 min. Cells were blocked for 30–60 min in Blocking buffer (PBS, 0.05% Triton X-100, 3% BSA) and incubated with primary antibodies (goat anti EloA [1:300 SCBT, R-19], anti Nopp140(NOLC1) [1:250 SCBT, (E-7): sc-374033]) in blocking buffer O/N at 4 °C. Coverslips were washed three times with wash buffer (PBS, 1% BSA, 0.05% Triton X-100) and incubated with secondary antibody conjugated to Alexa Fluor 568 or 488 for 60 min at room temperature, coverslips were washed three times with wash buffer, once with PBS, rinsed with water, and mounted on slides using DAPI-containing mounting medium. Images were acquired using the 60× oil objective of Nikon 90i Eclipse microscope equipped with Orca ER camera (Hamamatsu) and velocity imaging software (Perkin Elmer). Z-stacks were collected using the 0.2 μm spacing and analyzed using maximum intensity of each section using ImageJ Fiji software.

### Fluorescence recovery after photobleaching

Cells were grown on glass-bottomed fluorodish (WPI) in phenol-red free DMEM medium. Expression of mCherry-EloA was induced using doxycycline at the indicated concentration (50–100 ng/ml for 6 h). All images were acquired using a Ti-2 Eclipse microscope (Nikon). Imaging was performed at 37 °C using a temperature-controlled Tokai-Hit culture dish system. Incubation with Hoechst 33,342 (Molecular Probes, 1:5000) for 2 h was carried out for live-cell DNA staining. A square of 1.5 × 1.5 microns was imaged for 2 s at 1 frame/s before stimulation for photobleaching using a 561-nm laser at 10% power for 2 s. Two distinct time-lapse imaging protocols were used: a fast acquisition at 600 ms/frame for 20 s or a slow acquisition at 2 s/frame phase for 1 min. Images were background subtracted and fluorescence intensity evaluated at bleached areas using Fiji software. Measurements were normalized to values obtained before bleaching (t = 0) and compared with an unbleached area used as a reference. All recovery graphs show relative fluorescence intensity (RFI) as a function of time (s). For nucleoplasm FRAP experiment, squares of 1.5 × 1.5 microns were stimulated and imaged as described above.

### Generation of cell lines using lentiviral transduction

FLAG-EloA WT or FLAG-EloA skip IDR was cloned into a modified pTRIPZ vector (Dharmacon), described elsewhere (62). The skip IDR truncated construct contained three deletions in the IDR spanning amino acids (aa): 95–185, 236–335, 413–442, which was synthesized as a gBlocks Gene Fragments (IDT) and ligated to the sequence coding the C-terminal half of EloA. Constructs were transfected into HEK293T cells using TransIT lentiviral transfection reagent (Mirus) along with pCMV-dR8.91, which contains gag, pol, and rev genes and VSV-G envelope protein encoding pMD2.G plasmids. After 48 h, medium was collected and passed through 0.45 μm filter, and the medium was used to transduce 3T3 cells at low multiplicity of infection. After 2 days, transduced cells were selected using 1–2 μg/ml of puromycin. Expression of constructs at levels comparable with the endogenous levels of EloA was achieved through testing different concentration of doxycycline. Imaging was carried out on fixed cells as described in the immunofluorescence section above.

### Baculoviral expression and purification of proteins

For characterization of phase separation properties of EloA, FLAG-tagged EloA and FLAG-EloA in complex with EloB and EloC constructs were cloned in pFastBac1 baculovirus expression plasmid and purified and described previously (22). The construct for expression of FLAG-mEGFP was also described previously (62). For FLAG-mEGFP-EloA construct, EloA cDNA sequence was cloned downstream of FLAG-mEGFP in pFastBac1. Baculovirus was generated and used to infect Sf9 cells as described in detail elsewhere (22).

### Coimmunoprecipitation mass spectrometry (Co-IP/MS)

Wild-type and *EloA* null mESC cells growing on 100 mm gelatinized feeder plates were de-MEFed and 2 × 10^8^ cells were washed once in PBS, resuspended in lysis buffer (50 mM Tris pH 8.0, 0.25% NP-40, 50 mM NaCl, 1 mM EDTA, 5 mM MgCl_2_, 0.2 mM PMSF, 0.2 mM DTT, Roche protease inhibitor tablet) and left on ice for 10 min, dounced ten times, and spun down at 2850*g* for 5 min to separate nuclei from cytoplasmic extract. Nuclear extract was prepared as described elsewhere (63), by resuspending the nuclear pellet in low salt buffer (20 mM HEPES, pH 7.9, 25% glycerol, 1.5 mM MgCl_2_, 0.2 mM EDTA, 20 mM KCl, 0.2 mM DTT, 0.2 mM DTT, protease inhibitor) and while gently vortexing, equal volume of high salt buffer (20 mM HEPES, pH 7.9, 25% glycerol, 1.5 mM MgCl_2_, 0.2 mM EDTA 1.2 M KCl, 0.2 mM DTT, 0.2 mM DTT, protease inhibitor) was added to the resuspended nuclei. Nuclear extract was obtained by gentle continuous mixing in cold room for 30 min, followed by centrifugation at 20,000*g*. The concentration of the isolated nuclear extract was brought to 300 mM KCl using the low salt buffer and NP-40 was added to the extract to a final concentration of 0.05% before immunoprecipitation (IP). IP was carried out using 15 μg of goat anti EloA (SCBT, R-19) conjugated to magnetic protein G for 3 h at 4 °C. Beads were washed three times using wash buffer (20 mM HEPES, pH 7.9, 25% glycerol, 1.5 mM MgCl_2_, 0.2 mM EDTA, 300 mM KCl, 0.2 mM DTT, 0.2 mM DTT, protease inhibitor). Following the final washing step, the beads were eluted using 1× SDS gel-loading buffer and separated on a 10% SDS-PAGE gel 1/4 of the way down and stained using coomassie blue. Each immunoprecipitation was excised as 5 gel sections, minced, and digested in-gel using trypsin. Digested gel sections were dehydrated by treatment with acetonitrile and speed-Vac. Rehydration was carried out in 50 mM ammonium bicarbonate supplemented with 12.5 ng/μl of sequencing-grade trypsin (Promega) at 4 °C. Samples were incubated at 37 °C overnight. For peptide extraction, ammonium bicarbonate was removed and washed with a 50% acetonitrile,1% formic solution and dried in a speed-Vac. Dried samples were resuspended in HPLC solvent A (2.5% acetonitrile, 0.1% formic acid) and loaded into a reverse-phase HPLC column (containing 2.6 μm C18 spherical silica beads in afused silica capillary). Following formation of gradient, elution of peptides was carried out using increasing concentration of HPLC solvent B (97.5% acetonitrile, 0.1% formic acid). Electroionization was performed on the peptides before entering an LTQ Orbitrap Velos Pro ion trap mass spectrometer (Thermo Fisher Scientific). Detection, isolation, and fragmentation of the peptides were carried out to produce a tandem mass spectrum of fragment ions for each peptide. Sequest (Thermo Finnigan) was used for identification of peptide sequences. The false discovery rate (FDR) filter for peptide identification was set at 1% or less. Processing, preparation, and mass spectrometry analysis of the samples were carried out at Taplin Mass Spectrometry Facility (Harvard Medical School). Verification of EloA PAF1 interaction by immunoprecipitation Western Blot was carried out on 1 mg of nuclear extract using 1–3 μg of the following antibodies: Paf1 (Bethyl, A300-172A), CTR9 (Bethyl A301-395A), Leo1 (Bethyl, A300-175A), Parafibromin/CDC73, (Bethyl, A300-170A), EloA (R-19, Santa Cruz Biotechnology, sc-1557). Immunoblots were performed at a primary antibody concentration of 1:2000. Proteomic data analysis presented in Figure 4*A* was performed using the CRAPome (crapome.org) analysis pipeline (64).

### CRISPR/Cas9 generation of *Elongin A* knock out in NIH/3T3 cells

Guide RNA (gRNA) oligos targeting the 5ʹ untranslated region and translation initiation site of murine *EloA* gene were identified using crispr.mit.edu guide RNA design tool and cloned into pSpCas9(BB)-2A-Puro (PX459)V2.0 (addgene, Plasmid #62988) (65). Primer sequences for cloning both constructs are provided in Table S2. The day before transfection, 1.5 × 10^5^ 3T3 cells were plated in each well of a 6-well plate. On the next day, 1.25 μg of each plasmid (2.5 μg total) was transfected using 5 μl of jetPRIME (Polyplus) transfection reagent. After 24 h, transfected cells were selected using puromycin at a final concentration of 2 μg/ml and 48 h later, cells were washed once with PBS, trypsinized, counted, and diluted down to 5 cells/ml (0.5 cells/100 μl), and 100 μl was plated in each well of a 96-well plate (three plates total). One week later, wells with cells growing in them were expanded in a 12-well plate and harvested after reaching confluency. Harvested cells were frozen down and also used for verification of *EloA* KO by western blot using EloA (R-19, Santa Cruz Biotechnology, sc-1557) antibody.

### GREAT and wikipathways analysis

GREAT analysis (66) was carried out by uploading the differentially regulated enhancer BED files to the GREAT website (great.stanford.edu) and downloading the GO biological process list. Wikipathways (67) analysis was carried out by uploading the EloA enriched gene list to Enrichr (http://amp.pharm.mssm.edu/Enrichr) (68).

### Published data sources and processing

Published data were downloaded from GEO as follows: H3K4me1 (GSM747542) (69), H3K4me3 (GSM1385046) (70), H3K27Ac (GSM2586543) (71), H3K36me3 (GSM1873392) (72), H3K79me2 (GSM307151) (73), Oct4 (GSM1082340), Sox2 (GSM1082341), Nanog (GSM1082342), Med1(GSM1038259) (28), GRO-seq (GSE48895) (12).

FASTQ files were downloaded from GEO using sratools v2.9.1. All reads were processed as described before.

## Data availability

All sequencing data have been deposited to GEO under accession number: GSE151582. Mass spectrometry raw data are accessible under ProteomeXchange: PXD022138 and jPOSTrepo: JPST000951.

## Conflict of interest

The authors declare that they have no conflicts of interest with the contents of this article.
