# Supplementary material for: Elongin A associates with actively transcribed genes and modulates enhancer RNA levels with limited impact on transcription elongation rate in vivo
Source: J Biol Chem. 2020 Dec 24;296:100202. doi: 10.1074/jbc.RA120.015877 (PMC7948453; doi:10.1074/jbc.RA120.015877)
Supplement: Figures S1 to S3 and Tables S1 and S2 [file mmc1.pdf]

Supplementary Figure 1

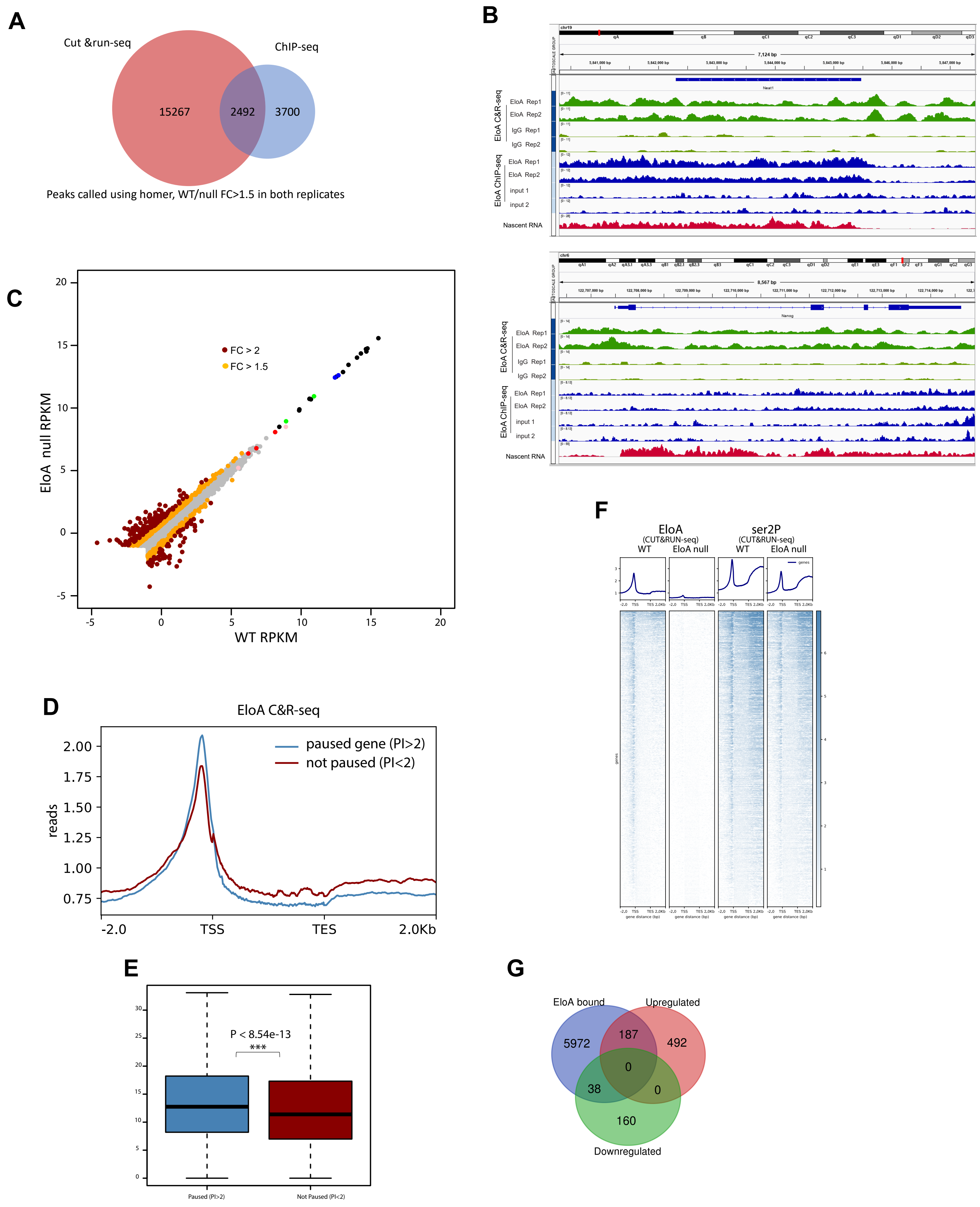

Supplementary Figure 2

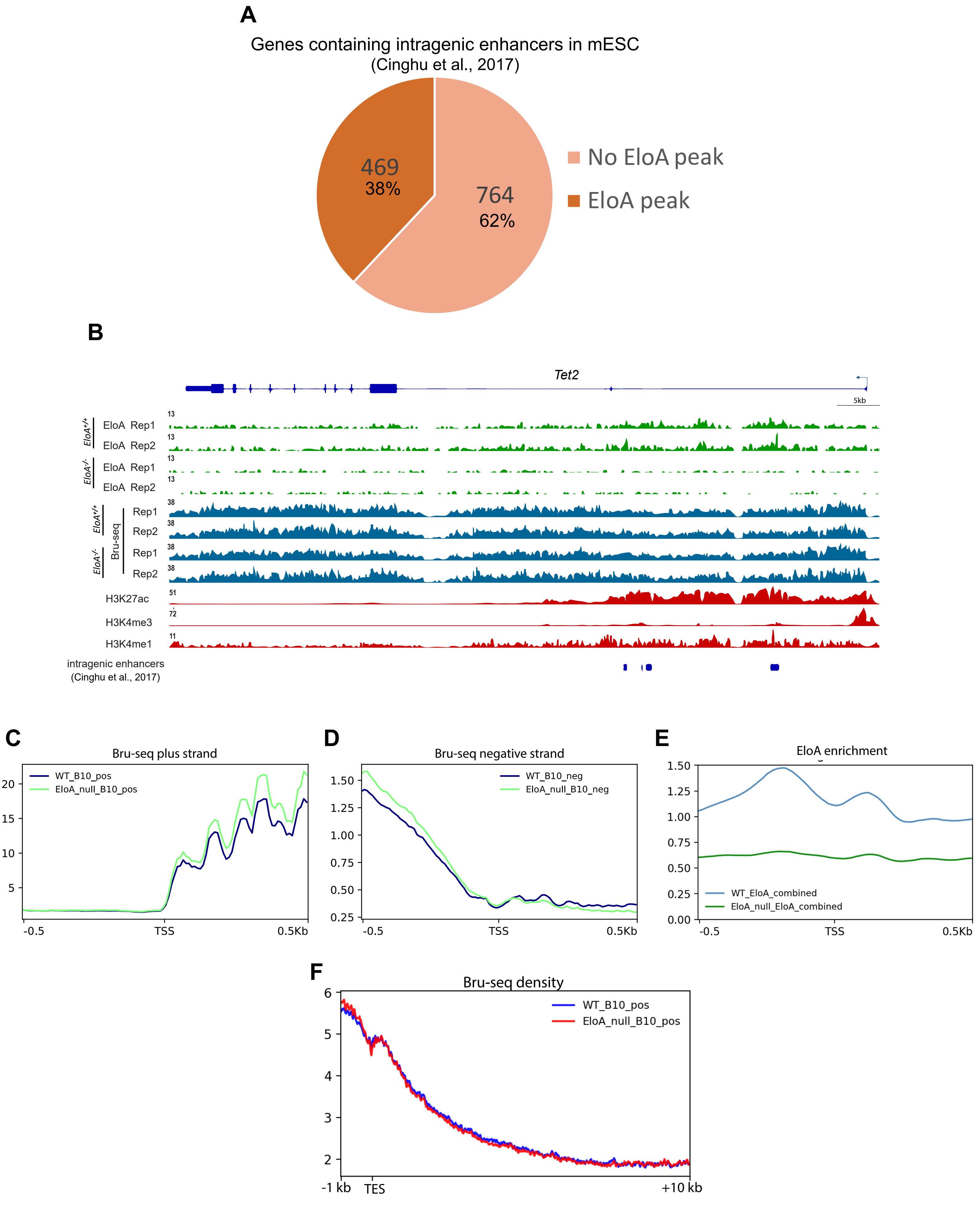

Supplementary Figure 3

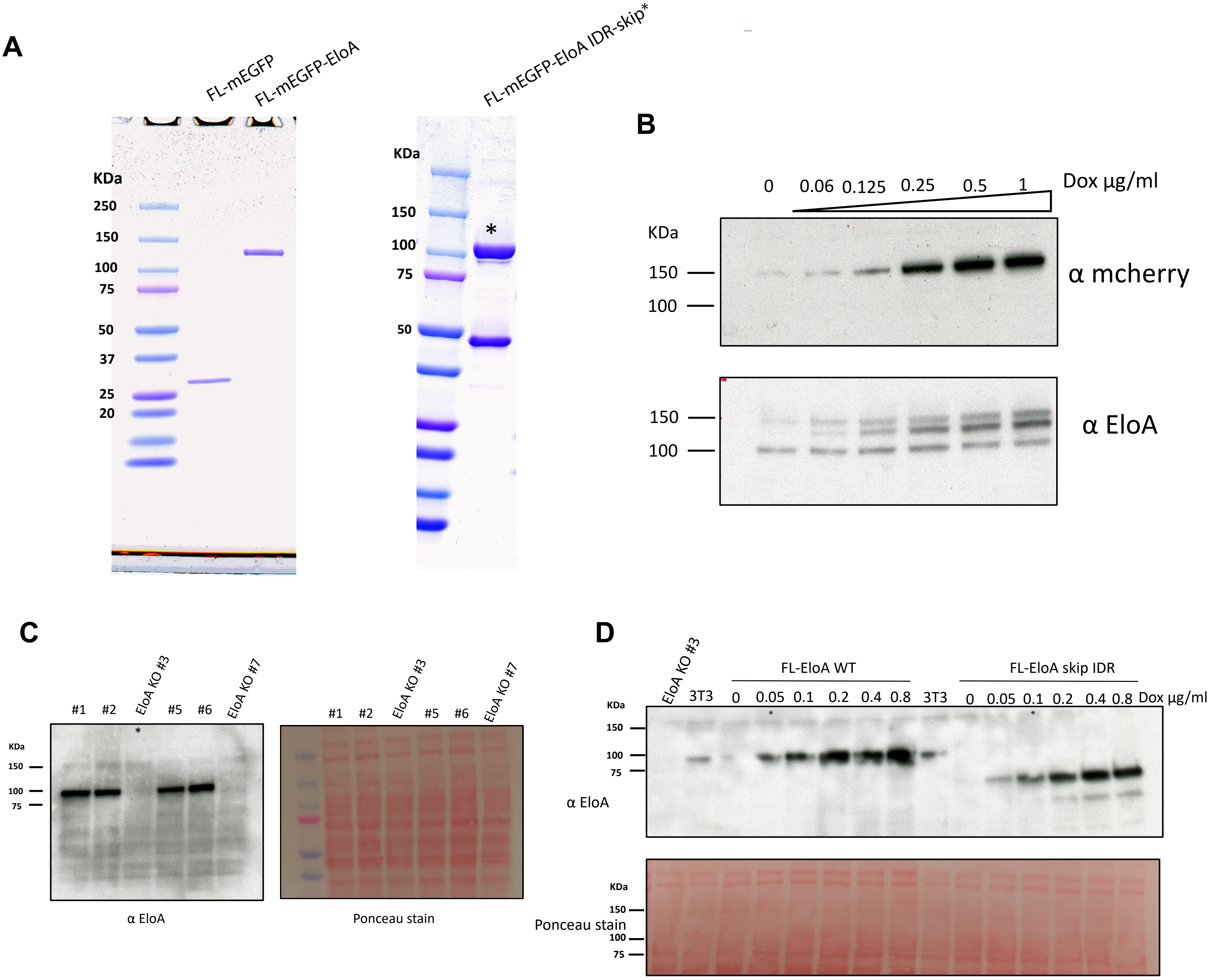

Supplementary Table 1: List of *in vitro* transcribed *Drosophila* Spike-in genes and primers used for generation of transcription templates (T7 promoter sequence highlighted in red).

Gene	Symbol	Flybase ID	T7-forward primer	reverse primer	Length (nt)
Usp	CG4380	FBgn0003964	GAATTAATACGACTCACTATAGGGA GCAAGCACCTCTGCTCTATT	GAAATCCCTGGACACGCTATT	440
Nep1	CG5905	FBgn0029843	GAATTAATACGACTCACTATAGGGA GACAAGAACAGCTCGGTGAA	TTGAACGCCGCACGTATAG	715
Rrp1	CG3178	FBgn0004584	GAATTAATACGACTCACTATAGGGA AGCTGAACCAGGGACAATAAG	TCCTTATCGGCGGATTTCTTC	430
RpA-70	CG9633	FBgn0010173	GAATTAATACGACTCACTATAGGGA AAGCAGCGGTAGACACAATAG	GTTGATCAGCAGTCGGTACTT	630
Piwi	CG6122	FBgn0004872	GAATTAATACGACTCACTATAGGGA TACACCCTGGATGATCGAACTA	ACTTGTTGCGAGACCAGATAAA	535
PHTF	CG3268	FBgn0028579	GAATTAATACGACTCACTATAGGGA CATAGACGCATGCTACGATCTC	GTGGTGCACTCTTGGTTACA	550
Tra1	CG33554	FBgn0053554	GAATTAATACGACTCACTATAGGGACCTCTGTTTGCCACTGTTCTA	TGGGTCCATCAACATTGGTAAA	400
Set1	CG40351	FBgn0040022	GAATTAATACGACTCACTATAGGGAGGAACGAGAATTTTTAGAGGAACA	TTTGGTTTTACCCTTTGAATTTGT	325
ESC	CG14941	FBgn0000588	GAATTAATACGACTCACTATAGGGATAACGAGCTGAAGTTCCATCCGCA	TGAAGCCGAATCTCACGAACCAGA	558
E(Z)	CG6502	FBgn0000629	GAATTAATACGACTCACTATAGGGAGTGGCGAGATCATATCCCAAGATG	TTTCACGCTCTATGCCCACGAACT	320
ERCC3	CG8019	FBgn0001179	GAATTAATACGACTCACTATAGGGACGAGGGAGAGGATTTCATTCAG	CACCACAAGGAAGCACAATAAC	460
GCM2	CG3858	FBgn0019809	GAATTAATACGACTCACTATAGGGA CCACCAGCACATCACCTATAA	GTATTTCCTGGTGCCGTAGTAG	575
PHO	CG17743	FBgn0002521	GAATTAATACGACTCACTATAGGGACCGGGATATCAGACGATGAATAC	CTCATGCAAGCCAATACGAAAT	465
Relish	CG11992	FBgn0014018	GAATTAATACGACTCACTATAGGGATTGAATGCGGACGGTGATAG	GAACAGAGCCGGTCGTAAAT	800
TFIIB	CG5193	FBgn0004915	GAATTAATACGACTCACTATAGGGAGCCAGAAGGAAATCGGTGATA	GGCAAATAAACGTAGTGTGCAA	475
Spt6	CG12225	FBgn0028982	GAATTAATACGACTCACTATAGGGA CAGTTGGAGGTGCCCTTTAT	CGTCTCCTTGGTCAGCATATC	2450
E2F	CG6376	FBgn0011766	GAATTAATACGACTCACTATAGGGAAATCCCAACCACCCACAATAG	AGCCAATGTCGTTGAAGAGAG	400
Taf1	CG17603	FBgn0010355	GAATTAATACGACTCACTATAGGGA AAACCAAGGAGGAGCAAGAG	GGAACAGGAATGGCGATACA	1450
Orc1	CG10667	FBgn0022772	GAATTAATACGACTCACTATAGGGA CAGGTGAAGTGGATCGGAAG	CCAGAGGCTCTTTGGATTTCT	1210
Caf1-55	CG4236	FBgn0263979	GAATTAATACGACTCACTATAGGGATCCCTACTCGGAGTTCATTCT	TCCTCGTCGTTGTAAACATTCT	410
Ecr	CG1765	FBgn0000546	GAATTAATACGACTCACTATAGGGA GCCGAGATGTGTTTCTCACTAA	AGGTGGTAGCTGAGGTTGTA	360
non-Bru RNA					
Mars	CG17064	FBgn0033845	GAATTAATACGACTCACTATAGGGA CCAACTATCTCAGTCCGTTTGT	GCTAATGTTTCCCTCCTCGTAG	765
Gpp	CG42803	FBgn0264495	GAATTAATACGACTCACTATAGGGA GCAAAGGAGATTGTGGGTAGA	TGGTAGAGGAGGAGGAAGATG	420
HsrΩ	CR31400	FBgn0001234	GAATTAATACGACTCACTATAGGGA GACTGCGAACTCGACTGAAATA	CAGCCAGGACGTTAGGTTAAA	960

Supplementary Table 2: Primers used for generation of NIH/3T3 EloA knockout cells by CRISPR-Cas9

primer	sequence
mEloA KO BF	CACC G CCAAGTTGTGGAGAAGCTCC
mEloA KO BR	AAAC GGA GCT TCT CCA CAA CTT GG C
mEloA KO CF	CACC G ACGCGAGCCCAGTTCCGGCG
mEloA KO CR	AAAC CGC CGG AAC TGG GCT CGC GT C

Supplementary Figure legends

Figure S1

A) Venn diagram showing the extent of overlap between ChIP-seq and C&R-seq data using (FC>1.5 over null) peak calling conditions. **B)** Comparison of ChIP-seq and C&R-seq IGV tracks at two genes showing enrichment of EloA at *Neat1* by both methods and low signal/background ratio for ChIP-seq EloA at *Nanog* (bottom panel). **C)** Scatter plot showing differential gene expression (DGE) between wild-type (WT) and *EloA* null mESCs as determined by nascent RNA sequencing using Bru-seq. Orange and red dots denote different fold-change cut offs (FC>1.5, FC>2, respectively). Pink, light red, green, blue and black dots at the center are *in vitro* transcribed *Drosophila* spike in control genes, list of fly spike in genes and primers used for generation of transcription templates is presented in supplementary table 1 (n=2 independent Bru-seq experiment). **D)** mESC GRO-seq data from Jonkers et al. (2014), was used to determine pausing index (Signal density near TSS -100 to +200 bp, over +300-+600 bp signal from TSS) as described by Henriques et al. (2018). Based on this data, genes were divided into paused (PI>2) and non-paused genes. Metagene plot shows higher level of EloA enrichment at the promoter/TSS region of paused genes. **E)** Box plot depicting higher levels of EloA at the TSS (+/- 500 bp) of paused (PI>2) genes in comparison to non-paused genes. *P*-values derived from Wilcoxon test. **F)** CUT&RUN-seq heat map showing enrichment levels of EloA and Ser2P in wild-type and EloA null mES cells. **G)** Venn diagram depicting the overlap between EloA-bound genes and genes that are upregulated or downregulated in EloA null mESCs (FC>1.5).

Figure S2 EloA is associated with intragenic enhancers.

A) Pie chart depicting the total number of genes that harbor intragenic enhancers (Cinghu *et al*, 2017), 469 (38%) of these genes showed enrichment and presence of EloA peaks at intragenic enhancers. **B)** IGV genome browser track showing enrichment of EloA at sites of intragenic enhancers (blue tracks, bottom row) at *Tet2.* **C)** Positive strand Bru-seq read density around the TSS (+/- 0.5 kb) showing higher levels of nascent transcripts, immediately after TSS in EloA null cells. **D)** same as C, but for negative strand nascent transcription, showing higher levels of upstream antisense transcription in null cells. **E)** Metagene profile of EloA CUT&RUN for the same set of genes displayed in C and D, showing enrichment of EloA at sites of divergent transcripiton. Y-axis scale denotes normalized reads. **F)** Loss of EloA does not lead to Pol II termination defect at the global level. Metagene line plots showing no effect on Bru-seq density (Pol II levels) during steady state transcription around TES and 10 kb downstream of TES. Y-axis scale: reads per million/bin.

Figure S3

A) Flag-mEGFP, Flag-mEGFP-EloA and Flag-mEGFP-EloA IDR-skip proteins were expressed in insect cells and affinity purified. SDS-PAGE gel stained with Coomassie blue, showing expression of mEGFP (29.1 KDa), mEGFP-EloA (116.3 KDa) and mEGFP-EloA IDR-skip (90.6 KDa). Protein band at around 50KDa in the IDR-skip preparation is uncharacterized. **B)** mCherry and EloA immunoblots showing expression of Dox-inducible mCherry-EloA in wild-type 3T3 fibroblast cells. Concentration range of 0.06-0.125 μg/mL of Dox was selected for expression of mCherry EloA for the FRAP assay described in Figure 5. **C)** Immunoblot validation of CRISPR/Cas9 generated null *EloA* 3T3 cell line. Clone #3 denoted with an asterisk, was selected for generation of wild-type and IDR mutant Elongin A cell lines. **D)** Dox-induction of FL-EloA and FL-EloA IDR-skip in EloA null background validating expression of both constructs. Concentrations denoted with asterisks were used for expression of each construct to a level comparable to the endogenous levels of EloA (3T3 lane).
